# Letter to the Editor Regarding the Article, “Development of a Nomogram to Predict Clinically Relevant Postoperative Pancreatic Fistula After Pancreaticoduodenectomy on the Basis of Visceral Fat Area and Magnetic Resonance Imaging: A Nomogram Incorporating the Inflammatory Burden Index and Drainage Fluid Amylase Levels”

**DOI:** 10.1245/s10434-024-15159-2

**Published:** 2024-03-28

**Authors:** Jiayue Zou, Xiaofeng Xue, Lei Qin

**Affiliations:** Department of Hepatobiliary and Pancreatic Surgery, Department of General Surgery, The First Affiliated Hospital of Soochow University, Suzhou, Jiangsu Province China

Dear editor,

We appreciate your suggestions in the article titled, “Development of a Nomogram to Predict Clinically Relevant Postoperative Pancreatic Fistula After Pancreaticoduodenectomy on the Basis of the Visceral Fat Area and Magnetic Resonance Imaging.”

Clinical data from 205 patients who underwent pancreaticoduodenectomy (PD) were collected and randomly divided into a training set and a testing set. Data were missing due to the retrospective design of the study. Data on the inflammatory burden index (IBI) were collected before surgery and on postoperative day (POD) 3. Data on drainage fluid amylase concentrations (DFAC) and serum amylase concentrations (SAC) were collected on PODs 1 and 3, and the ratio of drainage fluid to serum amylase concentrations (DFSAC) was calculated. The subgroups with clinically relevant postoperative pancreatic fistula (CR-POPF) were compared using the Mann-Whitney *U* test.

The results showed that preoperative IBI did not differ significantly between the patients with and those without CR-POPF (*P* = 0.713). In turn, significant between-group differences in IBI were observed on POD3 (*P* < 0.001), in DFSAC on POD1 (*P* = 0.004), in DFSAC on POD3 (*P* < 0.001), in DFAC on POD1 (*P* < 0.001), and in DFAC on POD3 (*P* < 0.001) (Table 1).

**Table 1:** Comparison of clinical variables between the patients with and those without clinically relevant postoperative pancreatic fistula (CR-POPF)

Variable	CR-POPF	non-CR-POPF	*P* Value
Preoperative IBI	2.559 (− 2.101 to 4.95)	2.514 (− 3.602 to 5.483)	0.713
IBI on POD3	5.612 (4.729 to 7.472)	5.172 (2.128 to 6.956)	<0.001
DFAC on POD1	7.342 (5.078 to 10.689)	5.911 (− 0.693 to 11.076)	<0.001
DFAC on POD3	7.496 (4.519 to 11.091)	5.332 (0.833 to 9.711)	<0.001
DFSAC on POD1	8.586 (0.172 to 4984.205)	2.805 (0.016 to 1457.567)	0.004
DFSAC on POD3	16.544 (0.303 to 547.974)	2.711 (0.0149 to 774.413)	<0.001

*IBI* inflammatory burden index; *POD* postoperative day; *DFAC* drainage fluid amylase concentrations; *DFSAC* ratio of drainage fluid to serum amylase concentrations

Patient characteristics did not differ significantly at baseline between the training and testing sets (*P* > 0.05; Table 2). Clinical variables were analyzed by uni- and multivariate logistic regression. Both IBI on POD3 and DFAC on POD3 were added to the predictive model such that the final model contained five variables: visceral fat area (VFA), pancreas-to-spleen signal intensity ratio (PSSI), main pancreatic duct diameter (MPDD), IBI on POD3, and DFAC on POD3 (Tables 3, 4). Among the patients with POPF, IBI on POD1, DFSAC on POD1, DFSAC on POD3, and DFAC on POD1 differed significantly but were not included in the model.

**Table 2:** Comparison of clinical variables between the training and testing sets

Variable	Training set	Testing set	*P* Value
Preoperative IBI	2.576 (− 2.102 to 5.483)	2.410 (− 3.602 to 5.418)	0.413
IBI-POD3	5.290 (3.638 to 6.956)	5.227 (2.128 to 7.472)	0.696
DFAC-POD1	6.304 (2.163 to 10.416)	6.624 (− 0.693 to 11.076)	0.816
DFAC-POD3	5.657 (1.482 to 11.091)	5.706 (0.833 to 10.994)	0.502
DFSAC-POD1	4.803 (0.027 to 636.053)	2.318 (0.016 to 4984.205)	0.189
DFSAC-POD3	4.060 (0.104 to 774.413)	3.362 (0.0149 to 547.974)	0.258

*IBI* inflammatory burden index; *POD* postoperative day; *DFAC* drainage fluid amylase concentrations; *DFSAC* ratio of drainage fluid to serum amylase concentrations

**Table 3:** Analysis of clinical variables by univariate logistic regression

Characteristics	OR	95 % CI	*P* Value
BMI	1.32	1.11–1.57	<0.001
DFAC on POD1	1.47	1.11–1.97	0.01
DFAC on POD3	1.81	1.35–2.43	<0.001
IBI on POD3	5.98	2.39–14.99	<0.001
MPDD	0.58	0.40–0.84	<0.001
PSSI	0	0–0.08	<0.001
VFA	1.01	1.01–1.02	<0.001

*OR* odds ratio; *CI* confidence interval; *BMI* body mass index; *DFAC* drainage fluid amylase concentrations; *POD* postoperative day; *IBI* inflammatory burden index; *MPDD* main pancreatic duct diameter; *PSSI* pancreas-to-spleen signal intensity ratio; *VFA* visceral fat area

**Table 4:** Analysis of risk factors by multivariate logistic regression

Variables	OR	95 % CI	*P* Value
DFAC on POD3	1.77	1.10–2.83	0.018
IBI on POD3	5.07	1.35–19.06	0.016
MPDD	0.47	0.27–0.84	0.010
PSSI	0	0–0.06	0.002
VFA	1.01	1.00–1.02	0.043

*OR* odds ratio; *CI* confidence interval; *DFAC* drainage fluid amylase concentrations; *POD* postoperative day; *IBI* inflammatory burden index; *MPDD* main pancreatic duct diameter; *PSSI* pancreas-to-spleen signal intensity ratio; *VFA* visceral fat area

The equation for the final five-variable model was

$$ \begin{aligned} X & = \left( {1.6990 \times {\text{IBI}} - {\text{POD}}3} \right) + \left( {0.5845 \, \times {\text{DFAC}} - {\text{POD}}3} \right) \\ & \quad + \left( {0.0122 \times {\text{VFA}}} \right){-}\left( {7.2302 \times {\text{PSSI}}} \right){-}\left( {0.7638 \times {\text{MPDD}}} \right). \\ \end{aligned} $$

**Fig. 1:**
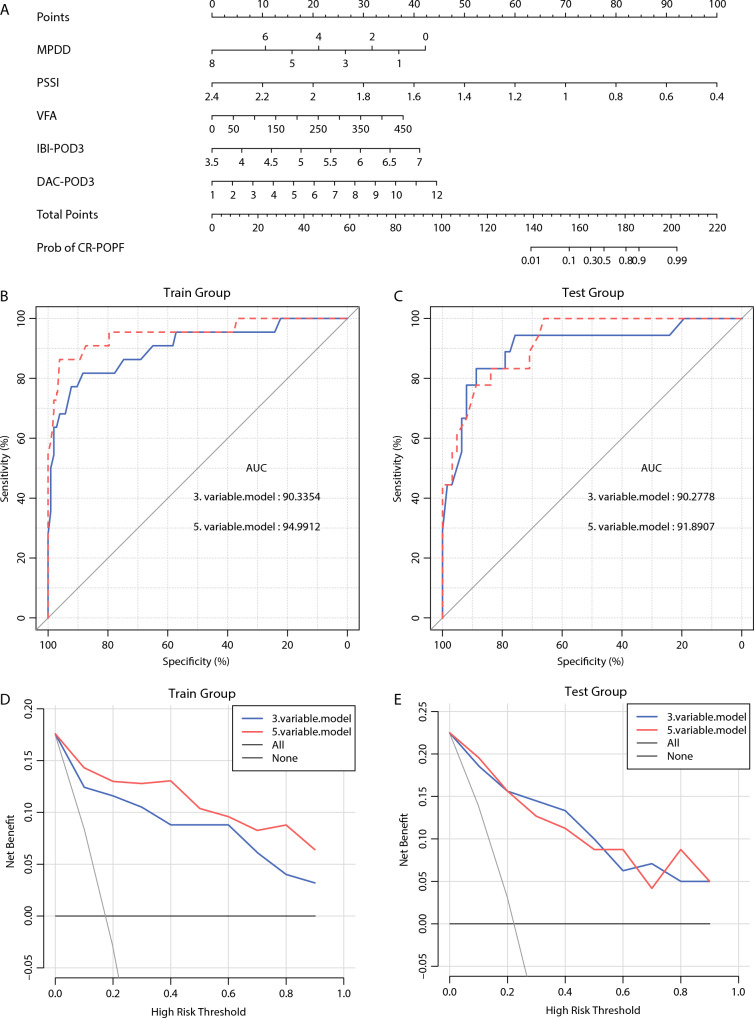
**A** The optimized prediction model in which five variables were scored, with higher total scores indicating a higher risk for occurrence of clinically relevant postoperative pancreatic fistula. **B, C** Comparison between the original three-variable model and the final five-variable model in the testing and training sets. The area under the curve (AUC) of the original and final models was 0.903 and 0.950 in the training set and 0.903 and 0.919 in the testing set. **D, E** Decision curve analysis of the two models in both datasets.

The nomogram was plotted using the rms package in R version 3.5.0 (Fig. 1A). The area under the curve (AUC) of the final model in the training set was 0.950, slightly higher than the AUC of the original three-variable model (0.903). The AUC of the final model in the testing set was 0.919, slightly higher than the AUC of the original model (0.903) (Fig. 1B, C).

Previous studies showed that DFAC on POD1 and POD3 determined the time when the drainage tubes were removal, consistent with our results. Decision curve analysis showed that the final model was superior to the original model in the training set (Fig. 1D, E). Integrated discrimination improvement (IDI) of the final model relative to the original model was 0.146 (95 % confidence interval [CI] 0.049–0.243; *P* = 0.003) in the training set and 0.021 in the testing set (95 % CI 0.058–0.099; *P* = 0.608) (Table 5).

**Table 5:** Integrated discrimination improvement (IDI) of the final model compared with the three-variable model

Dataset	IDI	95 % CI	*P* Value
Training	0.146	0.049 to 0.243	0.003
Testing	0.021	− 0.058 to 0.099	0.608

*CI* confidence interval

The risk of POPF can be predicted by DFAC and SAC.^1,2^ Inadequate blood supply to the anastomosis and local inflammation after PD are reflected by high SAC, impairing anastomotic healing and potentially leading to POFP. Consistent with our findings, high SAC and acute pancreatitis were shown to correlate with POPF.^3^ Additionally, DFSAC on POD3 can predict CR-POPF.^4^ Although DSFAC differed significantly between the patients with and those without CR-POPF, this parameter was not included in the model.

The IBI is used to assess the inflammatory status and survival in cancer patients. Higher IBI correlates with poorer outcomes and reduces patients’ quality of life and physical function.^5,6^ The neutrophil-to-lymphocyte ratio and C-reactive protein (CRP) are measures of systemic inflammation. Large surgical incisions and prolonged surgical time may affect local and systemic inflammation, further impairing the nutritional status and local healing and leading to delayed anastomotic growth and fistula development.^7^

The inclusion of DFAC on POD3 and IBI on POD3 increased the predictive ability of the final model. Thus, this model can guide the early removal of drainage tubes and postoperative recovery. However, this model could not predict CR-POPF preoperatively due to the inclusion of postoperative indicators.

A good model must make predictions as early and accurately as possible using simple data and easily accessible methods. The predictions made by the original model were based on preoperative variables. Moreover, although the predictive performance of the original model in the training set was slightly lower than that of the final model, the former identified patients at high risk of CR-POPF earlier for treatment and decision-making in the immediate preoperative period.

A larger multicenter study was not performed because of limited resources, reducing the generalizability of the findings. Nonetheless, we intend to conduct a large prospective clinical trial using the improved model to reduce the incidence of pancreatic fistula in our center.
